# AMOBH: Adaptive Multiobjective Black Hole Algorithm

**DOI:** 10.1155/2017/6153951

**Published:** 2017-11-23

**Authors:** Chong Wu, Tao Wu, Kaiyuan Fu, Yuan Zhu, Yongbo Li, Wangyong He, Shengwen Tang

**Affiliations:** ^1^School of Automation, China University of Geosciences, Wuhan 430074, China; ^2^Hubei Key Laboratory of Advanced Control and Intelligent Automation for Complex Systems, Wuhan 430074, China; ^3^State Key Laboratory of Water Resources and Hydropower Engineering Science, Wuhan University, Wuhan 430070, China

## Abstract

This paper proposes a new multiobjective evolutionary algorithm based on the black hole algorithm with a new individual density assessment (cell density), called “adaptive multiobjective black hole algorithm” (AMOBH). Cell density has the characteristics of low computational complexity and maintains a good balance of convergence and diversity of the Pareto front. The framework of AMOBH can be divided into three steps. Firstly, the Pareto front is mapped to a new objective space called parallel cell coordinate system. Then, to adjust the evolutionary strategies adaptively, Shannon entropy is employed to estimate the evolution status. At last, the cell density is combined with a dominance strength assessment called cell dominance to evaluate the fitness of solutions. Compared with the state-of-the-art methods SPEA-II, PESA-II, NSGA-II, and MOEA/D, experimental results show that AMOBH has a good performance in terms of convergence rate, population diversity, population convergence, subpopulation obtention of different Pareto regions, and time complexity to the latter in most cases.

## 1. Introduction

Most problems can be considered as multiobjective optimization problems (MOPs) [1, 2] in the fields of social production [3], engineering design [4], path planning [5], product design [6], motor design [7], mechanics design [8], and so forth. For example, to design a new toll plaza of a highway, at least two objectives should be considered, which are traffic efficiency and land cost. Hence, the study of MOPs is very meaningful in many research fields. Unlike the single-objective optimization problems (SOPs), there are multiple global optimum solutions which are called Pareto optimal solutions in MOPs. And objectives are often conflicting with each other [1]. Some traditional approaches use the weight function to transform MOPs into SOPs. However, they require some prior knowledge and can only get one solution of the Pareto optimal solution set.

Over the past decades, bioinspired computation and swarm intelligence based algorithms have been introduced into solving MOPs called evolutionary multiobjective optimization. Bioinspired computation is motivated by the natural and social behavioral phenomena and can start with a set of initial variables and then evolve to find multiple optima simultaneously [9]. Therefore, bioinspired computation is suitable for solving MOPs. The most popular multiobjective evolutionary algorithms (MOEAs) are Pareto dominance based algorithms [2], such as nondominated sorting genetic algorithm II (NSGA-II) [10] and strength Pareto evolutionary algorithm II (SPEA-II) [11]. Besides the criterion of Pareto dominance, they also adopted a diversity related secondary criterion to promote a good distribution of the solutions [12].

The BH algorithm was first proposed to solve the complex, hard optimization and clustering problem which is NP-hard [13]. It simulates the phenomenon of the black hole absorption. It has evolved from the PSO algorithm with new mechanisms. The BH algorithm searches the entire space of solutions (stars) and finds the global optimum solution (black hole). In the PSO algorithm, particles cannot disappear, which results in a premature convergence problem. To overcome the weakness of the PSO algorithm, in the BH algorithm, a star will be reborn randomly in the search space if it comes too close to the black hole. So, compared with the PSO algorithm, the BH algorithm has a better performance in avoiding the premature convergence phenomenon and a less time complexity than PSOs and GAs. What is more, the BH algorithm has only one controlling parameter which is the radius of the black hole.

A lot of MOEAs have two main problems: (1) high selection pressure or low selection pressure and (2) how to balance the diversity and convergence. Therefore, in this paper, we proposed a new multiobjective evolutionary algorithm which is based on the black hole algorithm (BH algorithm) [13] to solve these problems. A new individual density criterion called cell density is proposed to improve the convergence and diversity of Pareto optimal solutions. In this paper, the global optimum selection strategy is based on the Shannon entropy and will be adjusted adaptively. The Shannon entropy is used to analyze the status of evolution. To calculate the entropy, the approximate Pareto front is mapped to PCCS [14]. The elite mutation strategy referred to in [14, 15] is used for improving the local search ability of the proposed algorithm. We rank solutions in an archive based on the cell density and the concepts of strength of cell dominance [14, 16], respectively. And we introduce adaptive evolution strategies to adjust the elite learning rate and the global optimum selection strategy according to evolution status adaptively. A simulation using seven test problems with different degrees of difficulty which are ZDT1, ZDT3, ZDT4 [17], DLTZ2, DLTZ4, DLTZ5, and DLTZ7 [18], respectively, is demonstrated to investigate the scalability of AMOBH. The simulation results showed that AMOBH has a good performance in both diversity and convergence during solving different multiobjective optimization problems. Furthermore, we perform a comparison with four well-known MOEAs—SPEA-II [11], PESA-II [19], NSGA-II [10], and MOEA/D [20]—for evaluation. Besides, we use the metric called inverted generation distance [20] which can evaluate convergence and diversity at the same time to analyze the results. In order to further verify the diversity or uniformity of the results, we use the metric called spacing [21–23] to corroborate it. As will be shown in this study, the proposed algorithm outperforms the other four algorithms in terms of diversity and convergence in most cases. And it has a good time complexity.

## 2. Related Works and Motivation

After [24] first introduced vector evaluated genetic algorithms (VEGAs) to solve multiobjective optimization problems, a lot of MOEAs based on GAs have been proposed. Among them, the most representative algorithms are NSGA-II [10], SPEA-II [11], and MOEA/D [20]. And, recently, a lot of MOEAs based on GAs for solving many-objective problems were also proposed [1, 2, 12, 25, 26]. But all of them suffer from a slow convergence rate and a lot of time on generating new offspring, which are the main problems of GAs [27].

Compared with GAs, the particle swarm optimization (PSO) algorithm has the advantages of simplified formula, rather quick convergence rate, global optimization performance, fewer controlling parameters, and so forth. Based on the successful experience in the field of MOEAs using GAs, a multiobjective particle swarm optimization algorithm (MOPSO) was proposed soon [28]. Compared to some previous MOEAs, MOPSO has a faster convergence rate and it can get a rather satisfied and fully covered approximate Pareto front. Reference [29] introduced Shannon entropy [30] to analyze the MOPSO dynamics along the algorithm execution. Reference [14] introduced parallel coordinates in MOPSO to calculate entropy. However, the main problem of PSO algorithm is that it is easily trapped into a local optimum [31].

At present, a lot of MOEAs adopted the global optimum selection strategy based on the nondominated sorting [10, 11] or Pareto dominance [32] or hypervolume [33, 34] or niche [35, 36] and so on. But they all have some problems of high selection pressure or low selection pressure. In evolutionary multiobjective optimization, maintaining a good balance between convergence and diversity is particularly crucial to the performance of the evolutionary algorithms [37]. And most of the MOEAs face the problem of balancing the convergence and diversity. The evaluation of individual density is always based on one metric (such as Pareto dominance or crowding density), which results in the algorithm being unable to take into account both convergence and diversity [14].

Bearing these ideas and motivations in mind, an adaptive multiobjective black hole algorithm is proposed, investigated, and discussed in the following sections.

## 3. Definitions and Some Concepts

### 3.1. Multiobjective Optimization Problem

A minimum continuous unconstrained multiobjective optimization problem (in the optimization field, the maximization problems and minimization problems are dual problems) can be defined as follows:

(1) min Fx=f1x,f2x,…,fmxT,x∈Rn,

in which **x** = [*x*_1_, *x*_2_,…,*x*_*n*_]^*T*^ ∈ *C*, *n* is the number of decision variables, *f* is the objective function, *m* is the number of objectives, and *C* is an *n*-dimensional search space.

#### 3.1.1. Pareto Optimality

Normally, objectives are restricted or conflicting with each other [38], which results in the global optimum solution being not unique. There cannot be found a solution that is superior to all. But noninferior solutions exist; the so-called noninferior solutions are solutions that cannot be optimized for at least one objective; at the same time, other objectives will not deteriorate. And these solutions are called Pareto optimal.

Here are some definitions of Pareto optimality.

**Definition 1 (Pareto optimality).**

In search space, **x** and **x**^*∗*^ are the decision vectors; if the following conditions are satisfied:

(2) fix<fix∗,∀i∈M,  ∃x∈C,

**x**^*∗*^ is said to be Pareto optimal.

**Definition 2 (Pareto dominance).**

There are two objective vectors **u**, **v** ∈ *W*, where *W* is an *m*-dimensional objective space; if the following conditions are met:

(3) vi<ui,∃i∈M,vj≤uj,∀j∈M,  j≠i,

**v** is said to dominate **u**.

**Definition 3 (Pareto front).**

The set of all Pareto optimal solutions is called Pareto optimal set. The space composed of Pareto optimal objective vectors is called Pareto front.

### 3.2. Parallel Cell Coordinate System

Parallel coordinates are a common way of visualizing high-dimensional geometry and analyzing multivariate data [39]. Reference [16] proposed the GrEA which mapped the approximate Pareto front to grid coordinates. Inspired by these, [14] proposed a concept called parallel cell coordinate system (PCCS) which mapped the approximate Pareto front to a two-dimensional plane from Cartesian coordinates to integer coordinates. The formula of the transformation is defined as follows:

(4) $$\begin{array}{c} S_{n,m}=\left\{\begin{array}{cc} \left\lceil N\left(t\right)\frac{f_{n,m}-f_{m}^{min}}{f_{m}^{max}-f_{m}^{min}}\right\rceil , & f_{n,m}\neq f_{m}^{min};\\ 1, & f_{n,m}=f_{m}^{min}, \end{array}\right. \end{array}$$

where *S* is a two-dimensional plane grid composed of *N*(*t*) × *M* cells, ⌈*x*⌉ is a top integral function, *n* = 1,2,…, *N*(*t*), *N*(*t*) is the number of members in archive in the current iteration *t*, *m* = 1,2,…, *M*, *M* is the number of objectives to be optimized, *f*_*m*_^min^ and *f*_*m*_^max^ are the minimum and maximum of objective *m* in the current approximate Pareto optimal set, respectively, and *S*_*n*,*m*_ is the integral coordinate or integral label of *f*_*n*,*m*_ in PCCS.

To rank the solutions in archive, we need to introduce the following definitions [14, 16].

**Definition 4 (cell dominance).**

If *S*_*i*,*m*_ and *S*_*j*,*m*_  (*i* ≠ *j*) are integral coordinates of any two solutions in an archive, at the same time, the following conditions are satisfied:

(5) Si,m1≤Sj,m1,∀m1∈M,Si,m2<Sj,m2,∃m2∈M.

Solution *i* is said to cell-dominate solution *j*.

**Definition 5 (strength of cell dominance).**

The total number of solutions which are cell-dominated by solution *i* is said to be the strength of cell dominance of solution *i*.

### 3.3. Shannon Entropy

In 1949, Shannon et al. in their paper [30] introduced a concept which is called Shannon entropy, which was proposed as a measure of the amount of information that is missing before reception.

Shannon entropy *H* is defined as follows:

(6) $$\begin{array}{c} H\left(\;X\;\right)=-K_{e}\underset{x\in X}{\sum }p_{i}\left(\;x\;\right)\;log_{2}p_{i}\left(x\right), \end{array}$$

where *p*_*i*_(*x*) is the probability of occurrence of the *i*th possible value of the source symbol, *K*_*e*_ is a positive constant, and *X* is the collection of events *x*.

In this paper, we refer to [14, 29] to calculate *H* as follows:

(7) Ht=−Nt∑n=1Nt∑m=1Mpit log2pit;pit=c Numn,mtNtM,

in which *c* Num_*n*,*m*(*t*)_ is the number of objective vectors which are mapped to PCCS in the cell grid with indexes *n* and *m*, *N*(*t*) is the number of solutions in archive in the current iteration *t* and it will be changed with the change of the solution's number in archive, and *M* is the number of objectives.

The update formula of entropy is as follows:

(8) $$\begin{array}{c} \Delta H\left(\;t+1\;\right)=H\left(\;t+1\;\right)-H\left(\;t\;\right). \end{array}$$

It is a measure to evaluate the status of evolution. It was mentioned in [29] that entropy is able to capture the convergence rate of the algorithm. Entropy represents uniformity and diversity of approximate Pareto optimal solutions. Larger entropy means better uniformity and diversity. The evolution status update algorithm is as shown in Algorithm 1   [14], where  *N*(*t* + 1) and *N*(*t*) are the number of solutions in archive in iterations *t* + 1 and *t*, respectively, and *N* is the size of the archive. The initial entropy is *H*(0) and the initial delta entropy is Δ*H*(0) = *H*(0).

### 3.4. Black Hole Algorithm

Inspired by the black hole phenomenon, the BH algorithm was first put forward in [13].

In the BH algorithm, the best candidate at each iteration is selected as a black hole and all the other candidates form the normal stars. The creation of the black hole is not random and it is one of the real candidates of the population. Then, all the candidates move towards the black hole based on their current location and a random number.

The details of the BH algorithm are as follows. Like other population-based algorithms, in the proposed BH algorithm, a randomly generated population of candidate solutions (the stars) is placed in the search space of some problems or functions. After the initialization, the fitness values of the population are evaluated and the best candidate in the population, which has the best fitness value, is selected to be the initial black hole; at the same time, the rest form the normal stars. The black hole has the ability to absorb the stars that surround it. After initializing the black hole and stars, the black hole starts absorbing the stars around it and all the stars start moving towards the black hole. The absorption of stars by the black hole is formulated as follows:

(9) Xit+1=Xit+rand·Xbh−Xit,i=1,2,…,K.

Here, *X*_*i*_(*t*) and *X*_*i*_(*t* + 1) are the locations of the star *i* in iterations *t* and *t* + 1, respectively, *X*_bh_ is the location of the black hole in the search space, rand is a random number in the interval [0,1], and *K* is the number of stars (candidate solutions) or the size of population.

Every star (candidate solution) that crosses the event horizon of the black hole will be absorbed by the black hole. The radius of the event horizon in the BH algorithm is calculated using the following equation:

(10) $$\begin{array}{c} R=\left|\;\frac{F_{bh}}{\sum_{i=1}^{K}F_{i}}\;\right|, \end{array}$$

where *F*_bh_ is the fitness value of the black hole, *F*_*i*_ is the fitness value of the star *i*, *K* is the number of stars (candidate solutions), and *R* is the radius of the black hole.

When the distance between a candidate solution and the black hole (best candidate) is less than *R*, the candidate is collapsed and meanwhile a new candidate is created and distributed randomly in the search space. 

## 4. Adaptive Multiobjective Black Hole Algorithm

### 4.1. Multiobjective Black Hole Algorithm with Mutation

When the BH algorithm is extended from single-objective optimization to multiobjective optimization, the radius formula will be a little different. At the same time, the criterion of whether a star crosses the event horizon of the black hole or not is also changed. In this paper, the radius of the event horizon is defined as follows:

(11) $$\begin{array}{c} R_{j,k}=\left|\;\frac{Fb_{j,k}}{\sum_{i=1}^{K}F_{i,k}}\;\right|,\;k=1,2,\ldots ,M, \end{array}$$

in which *R*_*j*,*k*_ is the radius of the *k*th objective of the black hole *j*; *Fb*_*j*,*k*_ is the fitness value of the *k*th objective of the black hole *j*, *j* = 1,2,…, *N*_*b*_; *N*_*b*_ is the number of black holes; we set it to 2*M*; the reason will be explained in Section 4.4; and *F*_*i*,*k*_ is the fitness value of the *k*th objective of star *i*.

Therefore, the criterion of whether a star crosses the event horizon of the black hole or not is as follows: if ∃*i* ∈ 1,2,…, *K*, *j* ∈ 1,2,…, 2*M*, ∀*k* ∈ 1,2,…, *M*, |*F*_*i*,*k*_ − *Fb*_*j*,*k*_ | ≤*R*,

(12) Xit+1=Xboundmax−Xboundmin·rand+Xboundmin.

Here, *X*_boundmax_ is the upper bound of search space, *X*_boundmin_ is the lower bound of search space, rand is a random number in the interval [0,1], and *X*_*i*_(*t* + 1) is the location of the *i*th star in iteration *t* + 1.

#### 4.1.1. Star Mutation

Almost all PSO algorithms adopt the mutation to improve the prevention of precocious puberty and avoid the local optimum [40]. Similarly, this paper introduces this strategy to the BH algorithm. We introduce a random mutation into the stars' location update. If a random number is smaller than *p*, *p* is the mutation rate which will affect the degree of random oscillation, and then the star's location will be updated in search space randomly (the larger the value of *p*, the greater the random oscillation). This paper set *p* = 0.3.

The mutation of a star's location is shown as follows:

(13) Xit+1=Xboundmax−Xboundmin·rand+Xboundmin,i=1,2,…,K.

This formula will enhance the proposed algorithm's ability of global search and improve the searching accuracy by increasing random oscillation.

### 4.2. Cell Density

When the archive of approximate Pareto optimal solutions is full, both new solutions and old solutions are not superior to each other. To get well-distributed diversity, the algorithm needs to estimate the impact of replacing one old solution with a new solution on the individual density. This paper presents a new individual density concept called cell density in PCCS which uses the total number of solutions in the cell grid occupied by one solution as its individual density. The formula of cell density is defined as follows:

(14) $$\begin{array}{c} D_{i}=\frac{\sum_{m=1}^{M}\sum_{j=1}^{N_{S}}\left(S_{i,m}==S_{j,m}\right)}{V}, \end{array}$$

where *D*_*i*_ is the cell density of solution *i*, *M* is the number of objectives, *N*_*S*_ = *N*(*t*) + 1 is the number of solutions (when the archive is full, *N*_*S*_ = *N* + 1), *N* is the size of the archive, *S*_*i*,*m*_ and *S*_*j*,*m*_ are the integral labels of solution *i* and solution *j* of objective *m* in PCCS, respectively, and *V* is the area; in this paper, it is set to value 1.

**Figure 1:**
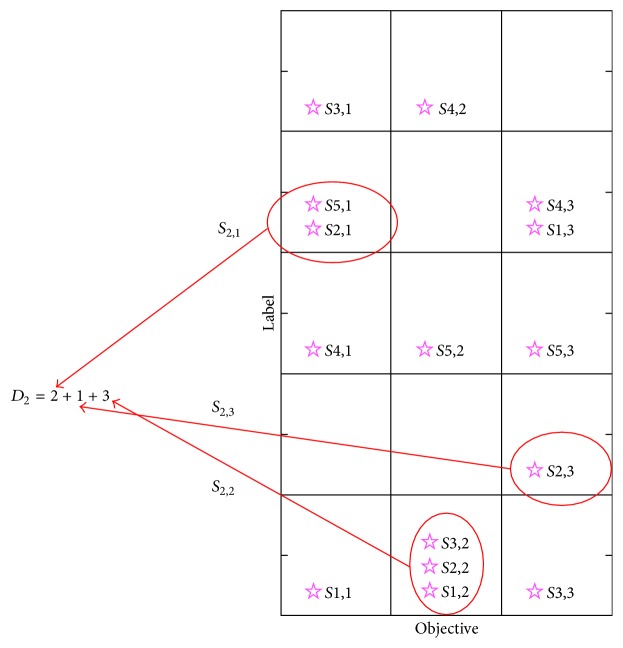
Approximate Pareto front in PCCS.

Take Figure 1 as an example; in this example, *M* = 3, *N* = 5. *S*1, 1 means solution 1 of objective 1. The cell density of all solutions is shown in Table 1.

As can be seen from Table 1, solution 1 and solution 2 have the max cell density, which is also proved by Figure 1. The example of how to compute the cell density of solution 2 is shown in Figure 1. If a new solution's cell density is lower than the max cell density of all old solutions, the new solution replaces one old solution of the max cell density and updates the archive. Otherwise, it should be refused.

### 4.3. Archive Maintenance Strategy

The algorithm begins with a fixed size (*N*) of archive. The archive is used for storing elite solutions. It needs to be updated when new solutions come in. The strategy of the archive's update is based on the cell density. When a new solution comes, there will be five situations as follows.

1. When the new solution and all old solutions are nondominant to each other and the archive is not full, accept the new solution into the archive.
2. When the new solution is inferior to some old solutions, refuse the new solution.
3. When the new solution is dominant to some old solutions, replace all inferior old solutions with the new solution.
4. When the new solution and all old solutions are nondominant to each other and the archive is full, we need to evaluate the cell density of the new solution and all old solutions. And if the cell density of the new solution is not the largest one, replace one old solution which has the largest cell density with the new solution.
5. When the new solution and all old solutions are nondominant to each other and the archive is full, we need to evaluate the cell density of the new solution and all old solutions. And if the cell density of the new solution is the largest one, refuse the new solution.

This strategy maintains the diversity of solutions in population evolution by pruning the large density solutions from the archive. It will maintain the high quality solutions and improve the convergence rate. The algorithm of the strategy is shown in Algorithm 2.

### 4.4. Adaptive Global Optimum Selection Strategy

In SOPs, the BH algorithm uses the global optimum solution as the black hole to update the location of stars (candidate solutions) and determine the direction of evolution. When the BH algorithm is extended to MOPs, the global optimum solution is not unique. If all the approximate Pareto optimal solutions are adopted as the black holes, this will cause a small selection pressure, hindering the effective promotion ability of the evolution process. What is more, it will slow down the convergence rate of the BH algorithm and make it premature and lose diversity. Accordingly, a reasonable strategy of global optimum selection which balances the diversification and intensification of the algorithm is necessary.

To get an approximate Pareto optimal solution set of good diversity and convergence, we need to select some nondominant solutions from the archive which can represent the diversity and convergence as the global optimum solutions. Because the convergence and diversity of solutions are generally conflicting, using different metrics to evaluate the convergence and diversity of the approximate Pareto optimal solutions in the archive, respectively, is very necessary.

This paper adopts the strength of the cell dominance as the convergence ranking metric and the cell density as the diversity ranking metric for selecting the global optimum solutions. It was mentioned in [14] that the whole population can share a common global optimum solution, but this will make the algorithm search with a single point which produces a high selection pressure, or each individual in the population uses different global optimum solutions, but this will lead to a scatter search which produces a low selection pressure. To control the selection pressure, we adopt the strategy of selecting global optimum solutions referred to in [14]. The strategy selects a total of 2*M* global optimum solutions from the archive for each iteration. And the selection process is based on the evolution status which can be adjusted with the change of evolution environment dynamically. Here, we give the algorithm of this strategy as shown in Algorithm 3.

### 4.5. Adaptive Elite Mutation Strategy

Inspired by [15], we adopt the strategy of elite mutation to increase the oscillation of local search. As mentioned earlier, all the solutions in the archive can be regarded as elite solutions. But here, we use this strategy to update the black holes (global optimal solutions' set which is got from Algorithm 3). Referred to in [14], the learning rate update algorithm is as shown in Algorithm 4, where Δ*H*(*t*) is the delta entropy of iteration *t*, *T* is the maximum of iteration, *t*/*T* is the update step size, and *l*_max_ and *l*_min_ are the maximum and minimum of learning rate, respectively; in this paper, we set *l*_max_ = 0.6 and *l*_min_ = 0.1.

The learning rate update algorithm will increase the oscillation of local search to skip the local optimum solutions quickly when the evolution status is “stagnation.” And if evolution status is “diversity,” the learning rate will decrease for improving the searching accuracy. It is a feedback control for balancing the diversification and intensification dynamically. And the elite mutation formula is as follows [15]:

(15) xit+1=ximax−ximin·Gaussian0,rand2+xit,

where *x*_*i*_(*t* + 1) and *x*_*i*_(*t*) are the *i*th decision variables of an elite solution in iterations *t* + 1 and *t*, respectively, *x*_*i*_^max^ is the maximum of the *i*th decision variable of all elite solutions, *x*_*i*_^min^ is the minimum of the *i*th decision variable of all elite solutions, rand is a random number uniformly distributed in the interval [0,1], and Gaussian(0, rand^2^) is a random number of a Gaussian function with a 0 mean and a standard deviation rand.

### 4.6. The Whole Algorithm

Through the previous analysis, the whole algorithm description of AMOBH is shown in Algorithm 5, where* APF* is the approximate Pareto optimal front,* APS* is the approximate Pareto optimal solutions, and* oV* is the objective vectors.

### 4.7. Computation Analysis

In Section 4.6, we can see that Algorithm 2 is in the innermost loop. And we need to calculate *N* + 1 solutions' cell density in the worst case when the archive is full. At the same time, to calculate cell density, the parallel cell coordinates of each solution need to be calculated. And this computation needs to be executed *M* × (*N* + 1) times in the worst case. So, the total computation of Algorithm 2 is *O*(*MN*^2^) which determines the computation of the full algorithm.

## 5. Results and Discussion

In this section, the paper uses seven functions with 2 or 3 objectives to be optimized of different degrees of difficulty to test the scalability of AMOBH. The dimensions of test functions range from 10 to 30. The seven test functions are ZDT1, ZDT3, ZDT4 [17], DLTZ2, DLTZ4, DLTZ5, and DLTZ7 [18]. These functions will test the proposed algorithm's performance in the different characteristics of the Pareto front: convexity, concaveness, discreteness, nonuniformity, and multimodality. All functions are to be optimized using AMOBH with iterations *T* = 1000, population size of stars *K* = 300, and archive size *N* = 50. To compare the performance of the proposed algorithm (AMOBH) with other multiobjective evolutionary algorithms, in this paper, we select four well-known multiobjective evolutionary algorithms (SPEA-II, PESA-II, NSGA-II, and MOEA/D) as the comparative algorithms. And the results of these functions' optimization and the performance analysis of comparative experiments are shown in the following sections. All experiments are carried out on a personal computer with an Intel Core 2.3 GHz CPU, 4 GB memory, and Windows 10 OS. The test functions are as follows.

ZDT1:

(16) min f1x=x1;min f2x=gx1−x1gx;s.t. gx=1+9∑i=2nxin−1,xi∈0,1,  i=1,2,…,n,  n=30.

ZDT3:

(17) min f1x=x1;min f2x=gx1−x1gx−x1sin⁡10πx1gx;s.t. gx=1+9∑i=2nxin−1,xi∈0,1,  i=1,2,…,n,  n=30.

ZDT4:

(18) min f1x=x1;min f2x=gx1−x1gx;s.t. gx=1+10n−1+∑i=2nxi2−10cos⁡4πxi,x1∈0,1,  xi∈−5,5,  i=2,3,…,n,  n=10.

DLTZ2:

(19) min f1x=1+gxcos⁡x1π2cos⁡x2π2;min f2x=1+gxcos⁡x1π2sin⁡x2π2;min f3x=1+gxsin⁡x1π2;s.t. gx=∑i=3nxi−0.52,xi∈0,1,  i=1,2,…,n,  n=10.

DLTZ4:

(20) min f1x=1+gxcos⁡x1απ2cos⁡x2απ2;min f2x=1+gxcos⁡x1απ2sin⁡x2απ2;min f3x=1+gxsin⁡x1απ2;s.t. gx=∑i=3nxi−0.52,xi∈0,1,  i=1,2,…,n,  n=10,  α=100.

DLTZ5:

(21) min f1x=1+gxcos⁡x1π2cos⁡π1+2gxx241+gx;min f2x=1+gxcos⁡x1π2sin⁡π1+2gxx241+gx;min f3x=1+gxsin⁡x1π2;s.t. gx=∑i=3nxi−0.52,xi∈0,1,  i=1,2,…,n,  n=10.

DLTZ7:

(22) min f1x=x1;min f2x=x2;min f3x=1+gx3−f1x1+gx1+sin⁡3πf1x−f2x1+gx1+sin⁡3πf2x;s.t. gx=1+9n−3∑i=3nxi,xi∈0,1,  i=1,2,…,n,  n=20.

### 5.1. Results of ZDT1 Optimization

**Figure 2:**
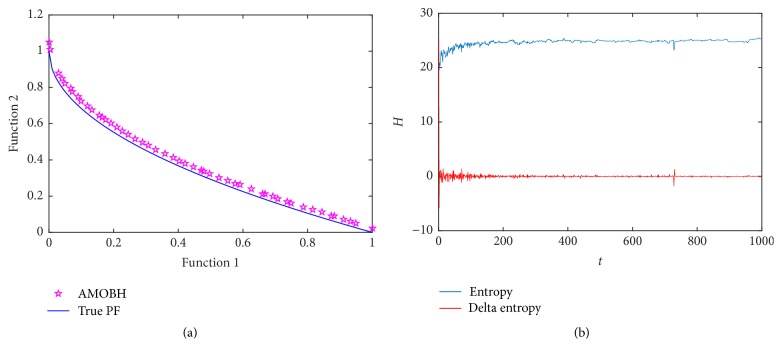
Simulation results of ZDT1. (a) The final approximate Pareto front of ZDT1. (b) Entropy during the AMOBH evolution for ZDT1.

The first optimization function is ZDT1. ZDT1 is very simple and is only used for the validation of feasibility of the proposed algorithm's application in multiobjective optimization problems. It has a convex Pareto optimal front.  Figure 2(a) illustrates the true and approximate Pareto front of ZDT1 in one of the experiments.  Figure 2(b) shows the entropy evolution during the optimization of ZDT1. As mentioned earlier, the entropy curve can be a measure to capture the convergence rate of the algorithm.

As can be seen from Figure 2(b), there is an ascending period which takes about 100 iterations. After that, the curve becomes smooth. Hence, AMOBH has a good convergence rate. It can be seen that the initial delta entropy is quite large. This is because the initial approximate Pareto optimal solutions that we select are *M* solutions which have the best fitness in at least one objective. As mentioned earlier, the entropy will change with the change of solutions' number in archive and the initial delta entropy is equal to the entropy in iteration 0. So, the initial cell density is very small, making the initial entropy and delta entropy quite large. And from Figure 2(a), we can see that AMOBH can get an approximate Pareto front which is quite close to the true Pareto front and has a good diversity.

### 5.2. Results of ZDT3 Optimization

**Figure 3:**
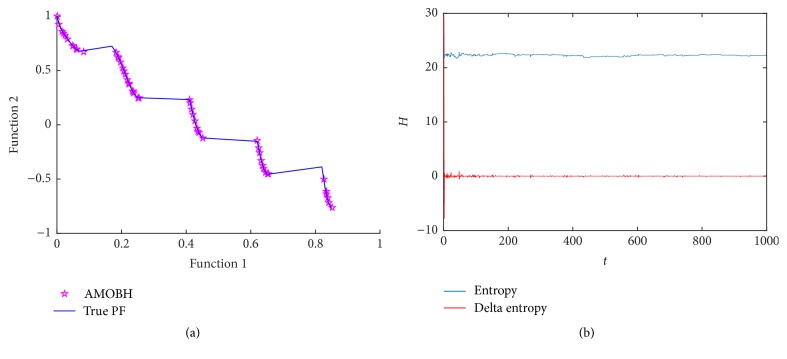
Simulation results of ZDT3. (a) The final approximate Pareto front of ZDT3. (b) Entropy during the AMOBH evolution for ZDT3.

To test the performance of the proposed algorithm in discontinuous problems, the second optimization function that we choose is ZDT3. It has a total of 30 decision variables. ZDT3 represents the discreteness features [17]. Its Pareto front has several noncontiguous convex parts.  Figure 3(a) illustrates the true and approximate Pareto front of ZDT3 in one of the experiments. From Figure 3(a), we can see that the convergence and diversity of the approximate Pareto front which is obtained by the proposed algorithm are quite good. Entropy evolution during the optimization of ZDT3 is represented in Figure 3(b). It can be seen that the convergence rate does not largely change. And it has been proved that the proposed algorithm has a good performance in some disconnected problems.

### 5.3. Results of ZDT4 Optimization

**Figure 4:**
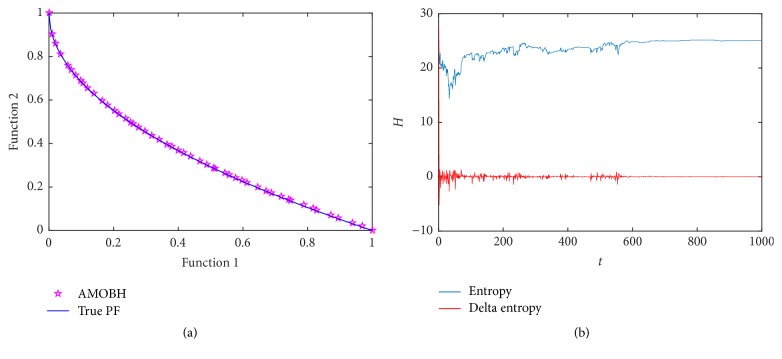
Simulation results of ZDT4. (a) The final approximate Pareto front of ZDT4. (b) Entropy during the AMOBH evolution for ZDT4.

The third optimization function is ZDT4. The Pareto front of ZDT4 is convex and it contains many local solutions. Hence, we use this function to test the proposed algorithm's ability to deal with multimodality.  Figure 4(a) illustrates Pareto front of ZDT4 in one of the experiments. In this function, we set the elite learning rate to 0.4 to get a better balance of diversity and convergence rate. We can see that the proposed algorithm converges very close to the true Pareto optimal front and it maintains a very diverse approximate Pareto optimal solution set. The entropy evolution during the optimization of ZDT4 is represented in Figure 4(b).

In Figure 4(b), we can see that the entropy curve is very different from that of previous functions' optimization. It has gone through several drastic changes. This is because it contains many local Pareto fronts. To skip these local Pareto fronts, many local solutions will be replaced from the archive which makes the drastic oscillation of the entropy curve. Therefore, it takes more time to reach convergence. It takes about 600 iterations to be stable. It is seen that the convergence rate of the proposed algorithm during solving some multimodal problems is not very ideal. It needs to be improved.

### 5.4. Results of DLTZ2 Optimization

**Figure 5:**
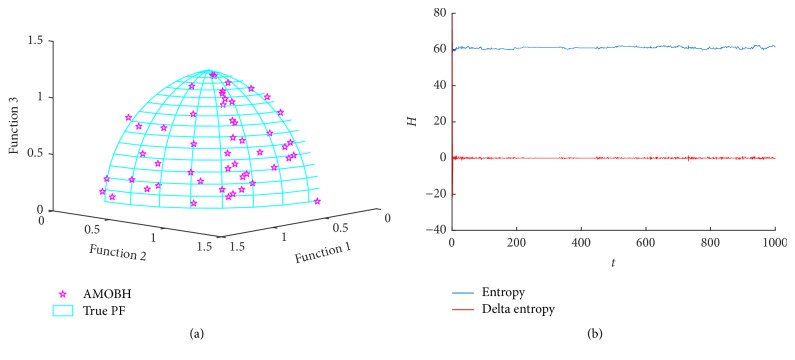
Simulation results of DLTZ2. (a) The final approximate Pareto front of DLTZ2. (b) Entropy during the AMOBH evolution for DLTZ2.

From this section, we extend test functions from two-objective problems to three-objective problems to test the scalability of AMOBH. The first three-objective optimization function is DLTZ2. It has 10 decision variables. The true Pareto front of DLTZ2 is a surface *F*_1_^2^ + *F*_2_^2^ + *F*_3_^2^ = 1, as the results illustrated in Figure 5(a). Figure 5(a) also illustrates the approximate Pareto front of DLTZ2 in one of the experiments. It can be seen that the proposed algorithm converges to an approximate Pareto front which is very close to the true Pareto front and has a good uniformity on the surface.  Figure 5(b) shows entropy evolution during the optimization of DLTZ2. We can see that the entropy curve is quite similar to ZDT3. Both of them have a very short dynastic initial period and a stable period with slight oscillation. And it has been proved that the proposed algorithm has a high convergence rate in some concave problems and a good scalability when the number of objectives is expanded from 2 to 3.

### 5.5. Results of DLTZ4 Optimization

**Figure 6:**
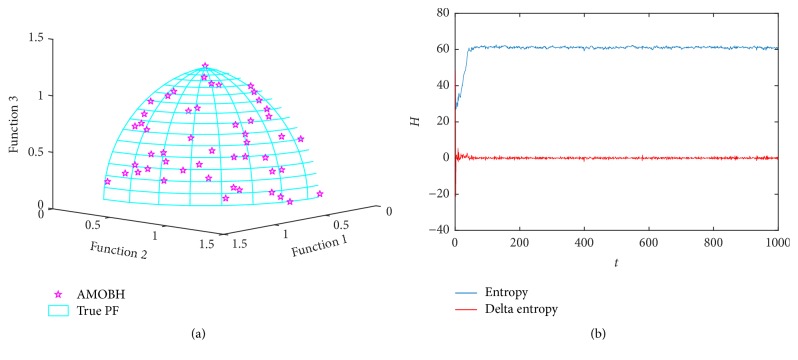
Simulation results of DLTZ4. (a) The final approximate Pareto front of DLTZ4. (b) Entropy during the AMOBH evolution for DLTZ4.

To further investigate the proposed algorithm's performance in three-objective optimization problems, the next optimization function we choose is DLTZ4. The function of DTLZ4 is very similar to DLTZ2, but all *x*_*i*_ in objectives of DLTZ2 are replaced by *x*_*i*_^*a*^ in objectives of DLTZ4. And *a* = 100. Therefore, the larger value of *α* makes the feature of Pareto front nonuniform. DLTZ4 is used to further test the proposed algorithm's ability to maintain a good distribution of solutions.  Figure 6(a) illustrates the true Pareto front and approximate Pareto front of DLTZ4 in one of the experiments. We can see that the true Pareto front of DLTZ4 is a surface same as DLTZ2. What is more, the proposed algorithm also converges to an approximate Pareto front which is very close to the true Pareto front and has a good distribution.  Figure 6(b) illustrates the entropy evolution during the optimization of DLTZ4. We can see the difference of entropy curve between DLTZ2 and DLTZ4. The initial period of DLTZ4 is an ascending curve and it takes much longer time to stabilize than DLTZ2. This is because of the nonuniform Pareto front of DLTZ4. However, it takes only 100 iterations to converge. These results confirm that the proposed algorithm has a quite high convergence rate and an ability to obtain good distribution solutions in some concave and nonuniform problems.

### 5.6. Results of DLTZ5 Optimization

**Figure 7:**
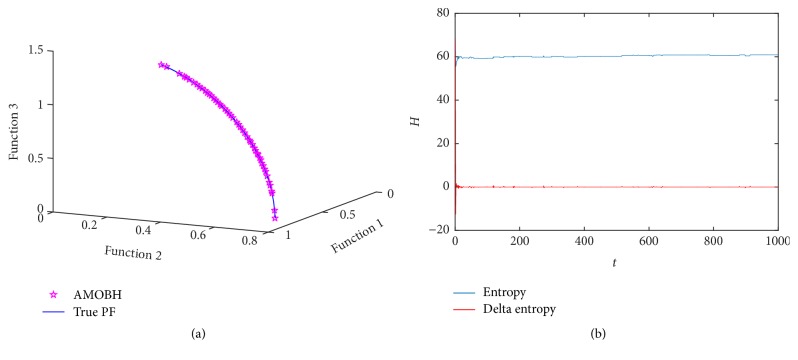
Simulation results of DLTZ5. (a) The final approximate Pareto front of DLTZ5. (b) Entropy during the AMOBH evolution for DLTZ5.

The next optimization function is DLTZ5. DLTZ5 is used to test the proposed algorithm's ability to converge to a degenerated curve.  Figure 7(a) illustrates the true and approximate Pareto front of DLTZ5 in one of the experiments. The true Pareto front of DLTZ5 is a part of a circle.  Figure 7(b) shows entropy evolution during the optimization of DLTZ5. It is seen that the entropy curve is very similar to DLTZ2, which means that the proposed algorithm has a good convergence rate and it can also be proved by the good convergent and uniform approximate Pareto front shown in Figure 7(a).

### 5.7. Results of DLTZ7 Optimization

**Figure 8:**
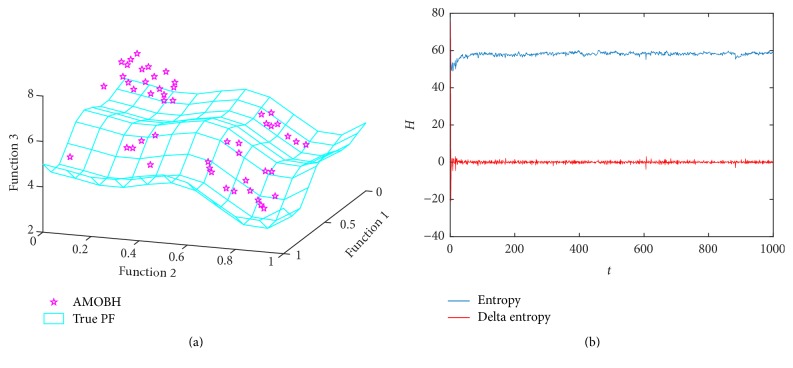
Simulation results of DLTZ7. (a) The final approximate Pareto front of DLTZ7. (b) Entropy during the AMOBH evolution for DLTZ7.

The final optimization function that we choose for three-objective optimization is DLTZ7; it has 4 disconnected Pareto front regions in search space. We use it to have a look at the performance of the proposed algorithm to obtain a subpopulation in different Pareto optimal regions.  Figure 8(a) illustrates the approximate Pareto front and true Pareto front of DLTZ7 in one of the experiments. As can be seen from Figure 8(a), the proposed algorithm gets an approximate Pareto front which covers the whole Pareto optimal regions and converges very close to the true Pareto front.  Figure 8(b) illustrates the entropy evolution during the optimization of DLTZ7. In the first 50 iterations, there is an obvious ascending phase, which means the diversity of the population has acute changes. This is because some new solutions replace some old solutions which have large cell density in the archive and make the entropy curve violently change. This also means that the algorithm frequently jumps out of one Pareto optimal region to another and keeps the diversity of population. And then, the entropy curve stabilizes in a certain range. As can be seen from these figures, the proposed algorithm has an ability to maintain the subpopulation in different Pareto optimal regions. It is also seen that the proposed algorithm can converge to an approximate Pareto front which is quite close to the true Pareto front, and it was further proved that AMOBH has a good scalability.

### 5.8. Comparative Experiment

In this section, we choose four well-known multiobjective evolutionary algorithms—SPEA-II, PESA-II, NSGA-II, and MOEA/D—for comparison with AMOBH. Before analyzing the results, we first describe the test metrics used for the experiments. In multiobjective optimization, there are mainly two goals to achieve regarding the obtained approximate Pareto optimal solution set [41]. The algorithm should converge as close to the true Pareto optimal front as possible and it should maintain as diverse an approximate Pareto optimal solution set as possible. Hence, the first test metric we choose is inverted generation distance (IGD) referred to in [20], which can evaluate convergence and diversity at the same time. Smaller IGD value means better diversity and convergence. The formula of IGD is shown as follows:

(23) IGD=1SN∑i=1SNDisti;Disti=minj=1ANsumm=1MfmSi−fmAjfmmax−fmmin2,

where |*S*_*N*_| is the sample number of the real Pareto optimal solutions, Dist_*i*_ is the minimum normalized Euclidean distance, |*A*_*N*_| is the number of the approximate Pareto optimal solutions, *f*_*m*_^max^ and *f*_*m*_^min^ are the maximum and minimum of objective *m* in the real Pareto optimal solution set, respectively, *S*_*i*_ is the sample *i* of the real Pareto optimal solution set, and *A*_*j*_ is the solution *j* of the approximate Pareto optimal solution set. The testing results and |*S*_*N*_| of each test function are shown in Table 2.

To further verify the uniformity of the results, the second test metric we choose is spacing [21–23]; the formula of spacing is as follows:

(24) Sp≅1AN−1sumi=1ANd¯−di2;d¯=sumi=1ANdiAN,

where *A*_*N*_ is the number of the approximate Pareto solutions and *d*_*i*_ is the Euclidean distance between the *i*th solution and its nearest neighbor in approximate Pareto solution set. Smaller *S*_*p*_ means better uniformity of the approximate Pareto solutions.

And to compare the time complexity of the proposed algorithm, we record the CPU runtime of all the comparative experiments and give the average CPU runtime in Table 2.

For the sake of fairness, all algorithms use the same max iteration *T* = 1000 and the same population size *N* = 50. For AMOBH, SPEA-II, PESA-II, and MOEA/D, the archive's size *K* = 50. In Tables 2 and 3, the Best, Worst, Mean, and STD represent the best, worst, mean, and standard deviation of IGD metric or spacing metric using the same algorithm which runs 25 times independently on the same test problem, respectively. And the boldface data are the best among all the data; the AT is the average CPU runtime.

**Figure 9:**
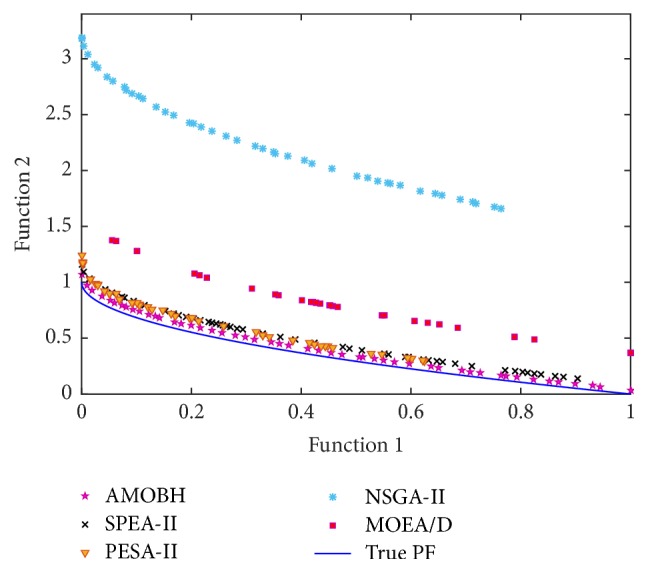
The approximate Pareto front of ZDT1 obtained by AMOBH, SPEA-II, PESA-II, NSGA-II, and MOEA/D, respectively, in one of the experiments.

**Figure 10:**
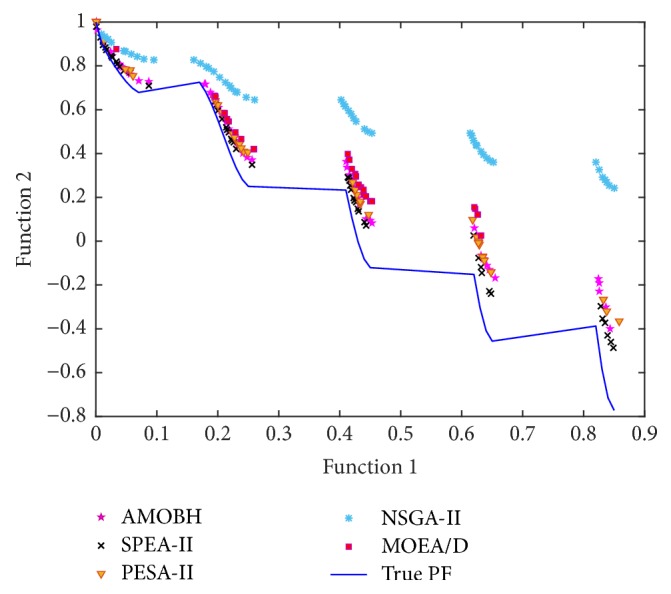
The approximate Pareto front of ZDT3 obtained by AMOBH, SPEA-II, PESA-II, NSGA-II, and MOEA/D, respectively, in one of the experiments.

**Figure 11:**
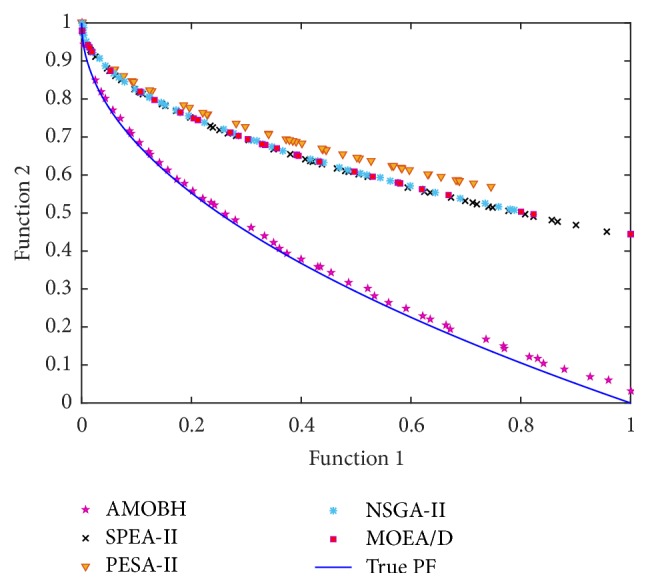
The approximate Pareto front of ZDT4 obtained by AMOBH, SPEA-II, PESA-II, NSGA-II, and MOEA/D, respectively, in one of the experiments.

**Figure 12:**
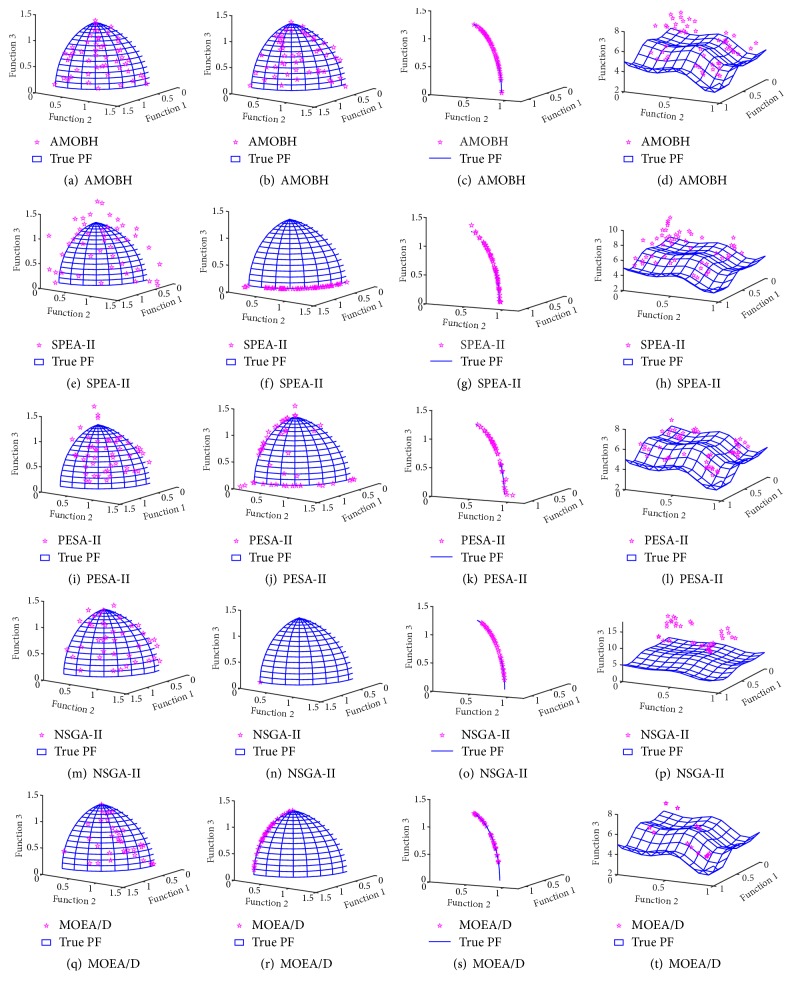
The approximate Pareto front of DLTZ2, DLTZ4, DLTZ5, and DLTZ7. (a)–(d). The approximate Pareto front obtained by AMOBH. (e)–(h). The approximate Pareto front obtained by SPEA-II. (i)–(l). The approximate Pareto front obtained by PESA-II. (m)–(p). The approximate Pareto front obtained by NSGA-II. (q)–(t). The approximate Pareto front obtained by MOEA/D.

In Table 2, we can see that the proposed algorithm achieves the best mean and STD values of IGD metric on the performance of all 7 problems. That is to say, the proposed algorithm has a good accuracy and stability on the performance of these problems. There is no significant difference between the proposed algorithm and SPEA-II on the performance of ZDT1 and ZDT3, which also can be seen in Figures 9 and 10. For the two objectives and multimodal problem, ZDT4, although NSGA-II gets the best STD value of IGD metric, there is no significant difference between NSGA-II and the proposed algorithm. However, the mean, best, and worst values of IGD metric on ZDT4 obtained by NSGA-II are significantly inferior to the proposed algorithm. Hence, the rank of performance on ZDT4 is AMOBH ≫ MOEA/D > SPEA-II > NAGA-II > PESA-II, and this is also evident from Figure 11. For the performance on nonuniform problem DLTZ4, the proposed algorithm gets the best balance of convergence and uniformity as Figures 12(b), 12(f), 12(j), 12(n), and 12(r) show. And for the performance on the disconnected multimodal problem DLTZ7, the proposed algorithm can converge to all 4 Pareto regions and has the best mean and STD values of IGD metric, which means the proposed algorithm can get a good balance of diversity and convergence on DLTZ7 although some solutions cannot converge to the real Pareto front, which also can be seen from Figure 12. According to previous analysis, it can be concluded that the proposed algorithm outperforms the other four algorithms on IGD metric, especially on the performance of ZDT4, DLTZ4, and DLTZ5. Comparison results show that the proposed algorithm can get a good balance of diversification and intensification in dealing with the problems of more objectives, nonuniformity, and multimodality.

Table 3 shows the results in terms of spacing metric of five algorithms. For the performance of ZDT1 and ZDT3, although the values of spacing metric obtained by the proposed algorithm are not the best, there is no significant difference between the proposed algorithm and the best one. It can be seen that PESA-II gets the best mean and STD values of spacing metric on ZDT1. SPEA-II gets the best mean value of spacing metric on ZDT3. Although NSGA-II gets the best STD value of spacing metric on ZDT3, there is no significant difference between NSGA-II and SPEA-II. However, the mean value of spacing metric on ZDT3 obtained by NSGA-II is significantly inferior to the SPEA-II. Hence, SPEA-II gets the best performance on ZDT3 in spacing metric. NSGA-II gets the best STD value of spacing metric on the performance of ZDT4 and the proposed algorithm is second to NSGA-II. However, the mean, best, and worst values of spacing metric on ZDT4 obtained by NSGA-II are significantly inferior to the proposed algorithm. That is to say, the proposed algorithm performs better on ZDT4 than NSGA-II in spacing metric. For the performance of DLTZ4, PESA-II gets the best mean value of spacing metric, but there is no significant difference between PESA-II and the proposed algorithm in the mean value of spacing metric, whereas the proposed algorithm is significantly superior to PESA-II in the STD value of spacing metric. That is to say, the proposed algorithm is more stable than PESA-II on the performance of DLTZ4 in spacing metric. The proposed algorithm gets the best mean and STD values of spacing metric on DLTZ2 and DLTZ5. Hence, the proposed algorithm is significantly superior to the other four algorithms on the performance of DLTZ2 and DLTZ5 which also can be seen in Figure 12. For the performance of DLTZ7, the rank of performance in mean spacing metric is PESA-II > SPEA-II > AMOBH ≫ MOEA/D > NAGA-II and the rank of performance in STD spacing metric is AMOBH > PESA-II > SPEA-II ≫ NAGA-II > MOEA/D. However, the difference between PESA-II and AMOBH is not so significant. This result has further corroborated the good scalability of the proposed algorithm. At the same time, in terms of the average CPU runtime, the proposed algorithm is significantly superior to the other four algorithms, as Table 2 shows.

## 6. Conclusions

This paper proposes a new multiobjective evolutionary algorithm based on an improved BH algorithm which adopts the mutation into the stars location update. In the proposed algorithm, we use Shannon entropy for adaptive evolution strategies' control. In order to get a uniform Pareto front distribution, we propose a new individual density concept which is called cell density to calculate the individual density of approximate Pareto optimal solutions in parallel cell coordinate system. In AMOBH, the entropy represents the uniformity and diversity of the approximate Pareto optimal solution set and it can be a measure to capture the convergence rate of the algorithm. Larger entropy means better uniformity and diversity. And the interpretation of cell density is to express the uniformity of approximate Pareto optimal solutions in parallel cell coordinate system. It is used for screening the solution which has max individual density to keep the diversity of the population. The simulations have confirmed its efficiency in screening. The optimization of seven functions was carried out to test the scalability of AMOBH. AMOBH gets a good balance of diversity and convergence in almost all test functions, although its convergence rate will be affected a lot on the performance of some multimodal problems. Through the comparative experiments with another four well-known multiobjective evolutionary algorithms, AMOBH outperforms the other four algorithms in most cases especially in some complex high-dimensional problems, which has further proved that AMOBH has a good scalability. And in terms of the time complexity, AMOBH is also much better than the other four algorithms. Hence, AMOBH is a new powerful multiobjective evolutionary algorithm.

To sum up, the BH algorithm can be used in multiobjective optimization problems like PSO algorithm and GA. Compared with some traditional multiobjective evolutionary algorithms, AMOBH has the advantages of solving the problems of higher dimensions and more objectives. And its time complexity is also quite good. In the future, we will be devoted to testing AMOBH in more complex multiobjective optimization problems like the many-objective optimization problems, comparing it with more multiobjective evolutionary algorithms and improving its convergence rate in some multimodal problems. What is more, we are considering to apply it to a DC motor parameters optimization problem.

## Figures and Tables

**Algorithm 1:**
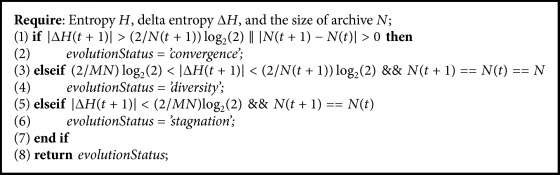
Evolution status update.

**Algorithm 2:**
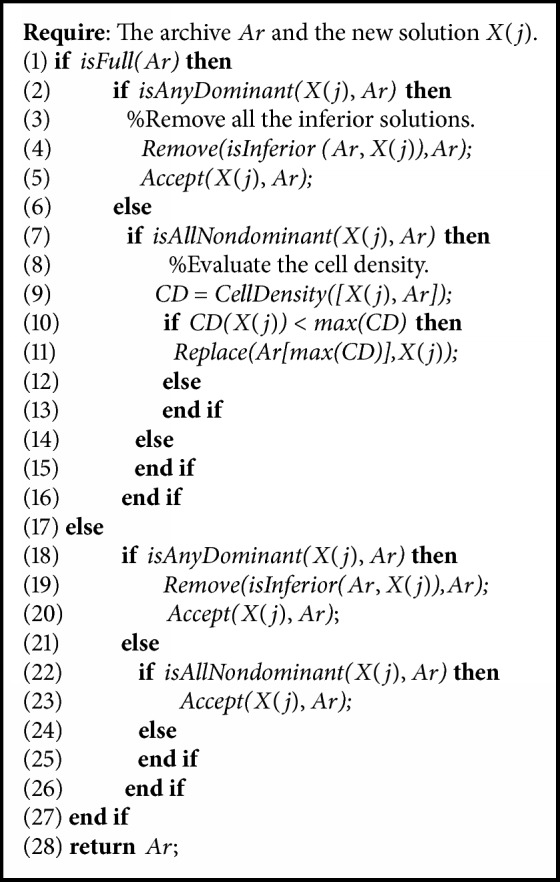
Archive maintenance.

**Algorithm 3:**
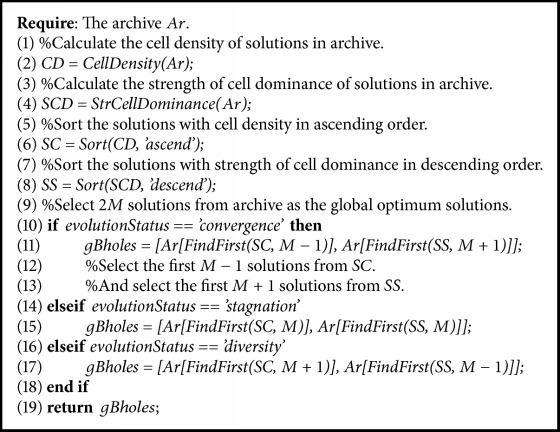
Adaptive global optimum selection.

**Algorithm 4:**
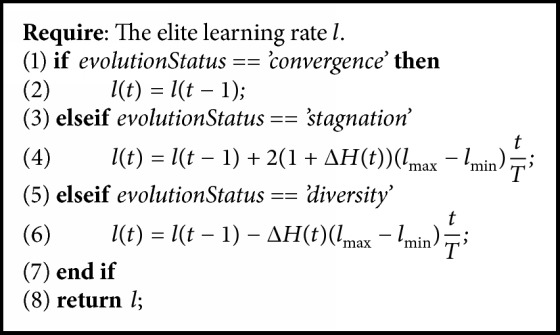
Elite learning rate update.

**Algorithm 5:**
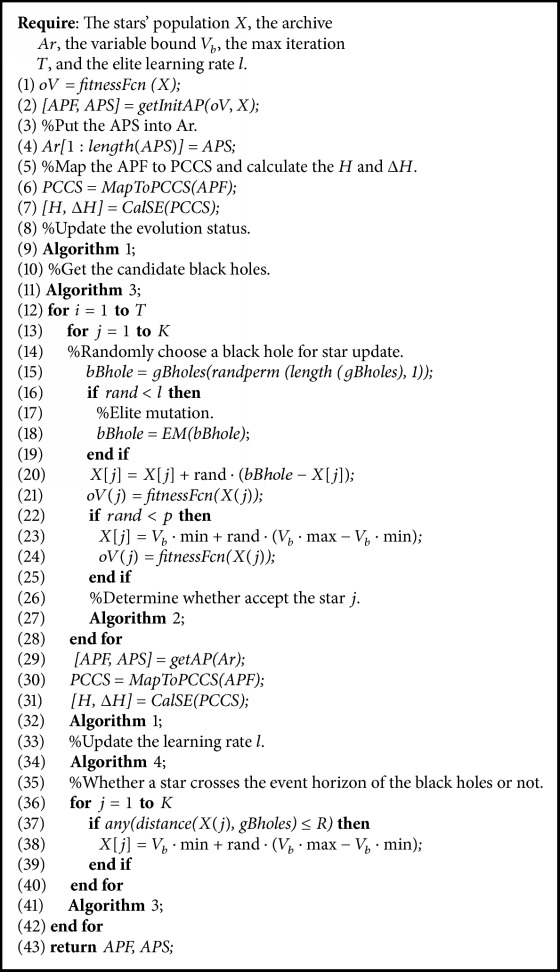
Adaptive multiobjective black hole algorithm.

**Table 1:** The cell density.

Solution 1	Solution 2	Solution 3	Solution 4	Solution 5
6	6	5	4	4

**Table 2:** Experimental results in terms of IGD and AT of five algorithms on seven test instances.

Test instance	SPEA-II	PESA-II	NSGA-II	MOEA/D	AMOBH	*S*_*N*_
ZDT1						
Mean	2.53*E* − 03	4.80*E* − 03	4.85*E* − 02	8.22*E* − 03	2.51**E** − 03	
Best	1.80*E* − 03	1.80*E* − 03	4.24*E* − 02	1.16**E** − 03	1.73*E* − 03	
Worst	3.50**E** − 03	1.22*E* − 02	6.20*E* − 02	4.63*E* − 02	3.60*E* − 03	1001
STD	4.88*E* − 04	2.43*E* − 03	4.56*E* − 03	1.14*E* − 02	4.71**E** − 04	
AT(s)	213	148	202	211	**33**	
ZDT3						
Mean	6.72*E* − 03	7.20*E* − 03	7.53*E* − 03	7.44*E* − 03	6.48**E** − 03	
Best	6.20*E* − 03	6.60*E* − 03	7.20*E* − 03	6.50*E* − 03	6.10**E** − 03	
Worst	7.50*E* − 03	7.70*E* − 03	8.50*E* − 03	1.02*E* − 02	6.77**E** − 03	1001
STD	3.40*E* − 04	3.62*E* − 04	2.49*E* − 04	9.01*E* − 04	1.48**E** − 04	
AT(s)	215	149	188	209	**34**	
ZDT4						
Mean	1.60*E* − 02	2.17*E* − 02	2.00*E* − 02	1.41*E* − 02	3.09**E** − 03	
Best	1.15*E* − 02	8.60*E* − 03	1.42*E* − 02	8.50*E* − 03	4.39**E** − 04	
Worst	2.39*E* − 02	3.43*E* − 02	2.37*E* − 02	1.79*E* − 02	8.80**E** − 03	501
STD	2.34*E* − 03	6.29*E* − 03	1.92**E** − 03	2.09*E* − 03	2.61*E* − 03	
AT(s)	212	172	199	214	**32**	
DLTZ2						
Mean	4.28*E* − 03	4.75*E* − 03	6.33*E* − 03	4.09*E* − 03	3.00**E** − 03	
Best	3.40*E* − 03	3.73*E* − 03	3.90*E* − 03	3.50*E* − 03	2.70**E** − 03	
Worst	5.20*E* − 03	6.20*E* − 03	2.18*E* − 02	4.80*E* − 03	3.30**E** − 03	1126
STD	4.56*E* − 04	6.45*E* − 04	3.58*E* − 03	4.20*E* − 04	1.27**E** − 04	
AT(s)	224	265	202	222	**38**	
DLTZ4						
Mean	7.84*E* − 03	5.20*E* − 03	4.02*E* − 02	1.75*E* − 02	3.12**E** − 03	
Best	3.60*E* − 03	3.60*E* − 03	3.11*E* − 02	3.60*E* − 03	2.80**E** − 03	
Worst	3.31*E* − 02	7.90*E* − 03	9.46*E* − 02	3.88*E* − 02	3.50**E** − 03	1126
STD	8.41*E* − 03	9.66*E* − 04	1.44*E* − 02	1.19*E* − 02	1.79**E** − 04	
AT(s)	217	275	201	220	**40**	
DLTZ5						
Mean	1.59*E* − 03	1.48*E* − 03	1.33*E* − 03	1.70*E* − 03	4.65**E** − 04	
Best	4.95*E* − 04	9.13*E* − 04	2.21**E** − 04	6.81*E* − 04	3.34*E* − 04	
Worst	1.70*E* − 02	2.50*E* − 03	4.60*E* − 03	2.62*E* − 03	6.04**E** − 04	1001
STD	3.23*E* − 03	4.03*E* − 04	1.17*E* − 03	5.25*E* − 04	7.20**E** − 05	
AT(s)	214	243	190	216	**34**	
DLTZ7						
Mean	4.39*E* − 03	4.47*E* − 03	3.89*E* − 02	1.92*E* − 02	4.30**E** − 03	
Best	3.10**E** − 03	3.30*E* − 03	3.14*E* − 02	9.40*E* − 03	3.70*E* − 03	
Worst	1.08*E* − 02	6.70*E* − 03	4.57*E* − 02	2.62*E* − 02	4.70**E** − 03	2601
STD	1.87*E* − 03	1.06*E* − 03	4.06*E* − 03	4.68*E* − 03	2.79**E** − 04	
AT(s)	216	252	197	166	**37**	

**Table 3:** Experimental results in terms of spacing of five algorithms on seven test instances.

Test instance	SPEA-II	PESA-II	NSGA-II	MOEA/D	AMOBH
ZDT1					
Mean	4.48*E* − 02	4.02**E** − 02	9.78*E* − 01	1.98*E* − 01	5.38*E* − 02
Best	2.74**E** − 02	3.06*E* − 02	8.05*E* − 01	3.13*E* − 02	4.06*E* − 02
Worst	6.30*E* − 02	6.14**E** − 02	1.23*E* + 00	1.09*E* + 00	6.98*E* − 02
STD	9.78*E* − 03	7.76**E** − 03	9.44*E* − 02	2.67*E* − 01	8.43*E* − 03
ZDT3					
Mean	1.07**E** − 02	1.98*E* − 02	3.67*E* − 02	4.57*E* − 02	1.40*E* − 02
Best	7.60**E** − 03	1.30*E* − 02	3.39*E* − 02	1.39*E* − 02	9.70*E* − 03
Worst	1.39**E** − 02	3.77*E* − 02	3.89*E* − 02	1.78*E* − 01	2.30*E* − 02
STD	1.58*E* − 03	6.07*E* − 03	1.31**E** − 03	3.19*E* − 02	2.97*E* − 03
ZDT4					
Mean	7.44*E* − 02	6.47*E* − 02	6.43*E* − 02	1.10*E* − 01	2.09**E** − 02
Best	5.21*E* − 02	1.45*E* − 02	5.86*E* − 02	4.97*E* − 02	1.16**E** − 02
Worst	9.93*E* − 02	1.03*E* − 01	7.46*E* − 02	8.47*E* − 01	4.09**E** − 02
STD	9.28*E* − 03	2.29*E* − 02	3.61**E** − 03	1.54*E* − 01	9.27*E* − 03
DLTZ2					
Mean	1.65*E* − 01	1.44*E* − 01	1.91*E* − 01	1.09*E* − 01	4.50**E** − 02
Best	8.06*E* − 02	5.83*E* − 02	6.04*E* − 02	2.93*E* − 02	2.23**E** − 02
Worst	2.76*E* − 01	3.33*E* − 01	6.99*E* − 01	3.40*E* − 01	7.68**E** − 02
STD	5.27*E* − 02	6.80*E* − 02	1.46*E* − 01	7.54*E* − 02	1.33**E** − 02
DLTZ4					
Mean	2.83*E* − 01	5.82**E** − 02	1.28*E* + 00	5.19*E* − 01	5.86*E* − 02
Best	1.13**E** − 02	1.96*E* − 02	1.17*E* + 00	2.82*E* − 02	2.54*E* − 02
Worst	1.18*E* + 00	2.21*E* − 01	2.54*E* + 00	1.25*E* + 00	8.30**E** − 02
STD	3.49*E* − 01	5.29*E* − 02	2.83*E* − 01	5.52*E* − 01	1.76**E** − 02
DLTZ5					
Mean	4.02*E* − 02	5.74*E* − 02	8.58*E* − 02	5.09*E* − 02	2.94**E** − 02
Best	1.00**E** − 02	1.80*E* − 02	1.01*E* − 02	1.36*E* − 02	1.09*E* − 02
Worst	1.30*E* − 01	1.85*E* − 01	3.06*E* − 01	1.77*E* − 01	3.93**E** − 02
STD	3.54*E* − 02	3.98*E* − 02	8.27*E* − 02	3.94*E* − 02	8.70**E** − 03
DLTZ7					
Mean	1.76*E* − 01	1.65**E** − 01	1.59*E* + 00	9.18*E* − 01	2.13*E* − 01
Best	1.22*E* − 01	1.20**E** − 01	1.32*E* + 00	4.55*E* − 01	1.65*E* − 01
Worst	5.12*E* − 01	2.17**E** − 01	1.84*E* + 00	1.20*E* + 00	2.46*E* − 01
STD	9.74*E* − 02	2.37*E* − 02	1.45*E* − 01	1.89*E* − 01	1.93**E** − 02

## References

[1] Gong D., Miao Z., Sun J. A memetic algorithm for multi-objective optimization problems with interval parameters.

[2] Gong D., Sun F., Sun J., Sun X. (2017). Set-based many-objective optimization guided by a preferred region. *Neurocomputing*.

[3] Hoffenson S., Dagman A., Söderberg R. (2013). A multi-objective tolerance optimization approach for economic, ecological, and social sustainability. *Re-Engineering Manufacturing for Sustainability*.

[4] Sanchis J., Martnez M. A., Blasco X., Reynoso-Meza G. (2010). Modelling preferences in multi-objective engineering design. *Engineering Applications of Artificial Intelligence*.

[5] Zhang Y., Gong D.-W., Zhang J.-H. (2013). Robot path planning in uncertain environment using multi-objective particle swarm optimization. *Neurocomputing*.

[6] Wasley N. S., Lewis P. K., Mattson C. A., Ottosson H. J. (2016). Experimenting with concepts from modular product design and multi-objective optimization to benefit people living in poverty. *Development Engineering*.

[7] Qiu H., Duan H. (2015). Multi-objective pigeon-inspired optimization for brushless direct current motor parameter design. *Science China Technological Sciences*.

[8] Bharti P. S., Maheshwari S., Sharma C. (2012). Multi-objective optimization of die-sinking electric discharge machining. *Applied Mechanics and Materials*.

[9] Zhou Ma., Zhang L.-b., Zhou C.-g., Ma M., Liu X.-h. (2004). Solutions of multi-objective optimization problems based on particle swarm optimization. *Journal of Computer Research and Development*.

[10] Deb K., Pratap A., Agarwal S., Meyarivan T. (2002). A fast and elitist multiobjective genetic algorithm: NSGA-II. *IEEE Transactions on Evolutionary Computation*.

[11] Zitzler E., Laumanns M., Thiele L. (2001). *SPEA-II: Improving the Strength Pareto Evolutionary Algorithm*.

[12] Liu Y., Gong D., Sun J., Jin Y. (2017). A many-objective evolutionary algorithm using a one-by-one selection strategy. *IEEE Transactions on Cybernetics*.

[13] Zhang J., Liu K., Tan Y., He X. Random black hole particle swarm optimization and its application.

[14] Hu W., Yen G. G., Zhang X. (2014). Multiobjective particle swarm optimization based on Pareto entropy. *Journal of Software *.

[15] Zhan Z.-H., Zhang J., Li Y., Chung H. S.-H. (2009). Adaptive particle swarm optimization. *IEEE Transactions on Systems, Man, and Cybernetics, Part B: Cybernetics*.

[16] Yang S., Li M., Liu X., Zheng J. (2013). A grid-based evolutionary algorithm for many-objective optimization. *IEEE Transactions on Evolutionary Computation*.

[17] Zitzler E., Deb K., Thiele L. (2006). Comparison of multiobjective evolutionary algorithms: Empirical results. *Evolutionary Computation*.

[18] Deb K., Thiele L., Laumanns M., Zitzler E. Scalable multi-objective optimization test problems.

[19] Corne D. W., Jerram N. R., Knowles J. D., Oates M. J. PESA-II: region-based selection in evolutionary multiobjective optimization.

[20] Zhang Q., Li H. (2007). MOEA/D: a multiobjective evolutionary algorithm based on decomposition. *IEEE Transactions on Evolutionary Computation*.

[21] Schott J. R. (1995). Fault tolerant design using single and multicriteria genetic algorithm optimization. *Cellular Immunology*.

[22] Veldhuizen D. A. V., Lamont G. B. On measuring multiobjective evolutionary algorithm performance.

[23] DongDong Y., LiCheng J., MaoGuo G., Hang Y. (2010). Clone selection algorithm to solve preference multi-objective optimization. *Journal of Software*.

[24] Schaffer J. D. Multiple objective optimization with vector evaluated genetic algorithms.

[25] Tang L., Wang X. (2013). A hybrid multiobjective evolutionary algorithm for multiobjective optimization problems. *IEEE Transactions on Evolutionary Computation*.

[26] Gong D., Wang G., Sun X., Han Y. (2015). A set-based genetic algorithm for solving the many-objective optimization problem. *Soft Computing*.

[27] Niu B., Wang H., Wang J., Tan L. (2013). Multi-objective bacterial foraging optimization. *Neurocomputing*.

[28] Coello Coello C. A., Lechuga M. S. MOPSO: a proposal for multiple objective particle swarm optimization.

[29] Pires E. J. S., Machado J. A. T., de Moura Oliveira P. B. (2013). Entropy diversity in multi-objective particle swarm optimization. *Entropy*.

[30] Shannon C. E., Weaver W., Wiener N. (1949). *The Mathematical Theory of Communication*.

[31] Ling S. H., Iu H. H. C., Leung F. H. F., Chan K. Y. (2008). Improved hybrid particle swarm optimized wavelet neural network for modeling the development of fluid dispensing for electronic packaging. *IEEE Transactions on Industrial Electronics*.

[32] Ganguly S., Sahoo N. C., Das D. (2013). Multi-objective particle swarm optimization based on fuzzy-Pareto-dominance for possibilistic planning of electrical distribution systems incorporating distributed generation. *Fuzzy Sets and Systems*.

[33] Bader J., Brockhoff D., Welten S., Zitzler E. (2009). On using populations of sets in multiobjective optimization. *EMO*.

[34] García I. C., Coello C. A. C., Arias-Montaño A. MOPSOhv: A new hypervolume-based multi-objective particle swarm optimizer.

[35] Liu W., Xie C., Wen J., Wang J., Wang W. (2013). Optimization of transmission network maintenance scheduling based on niche multi-objective particle swarm algorithm. *Zhongguo Dianji Gongcheng Xuebao/Proceedings of the Chinese Society of Electrical Engineering*.

[36] Bi X., Wang C. (2017). A niche-elimination operation based NSGA-III algorithm for many-objective optimization. *Applied Intelligence*.

[37] Cheng R., Jin Y., Olhofer M., Sendhoff B. (2016). A Reference Vector Guided Evolutionary Algorithm for Many-Objective Optimization. *IEEE Transactions on Evolutionary Computation*.

[38] Pan A., Tian H., Wang L., Wu Q. (2016). A decomposition-based unified evolutionary algorithm for many-objective problems using particle swarm optimization. *Mathematical Problems in Engineering*.

[39] Inselberg A. (2009). *Parallel Coordinates: Visual Multidimensional Geometry and its Applications*.

[40] Hu W., Li Z. S. (2007). A simpler and more effective particle swarm optimization algorithm. *Journal of Software *.

[41] Deb K. (2001). *Multiobjective Optimization Using Evolutionary Algorithms: An Introduction*.

